# Notes and News

**Published:** 1898-01-29

**Authors:** 


### NOTES AND NEWS.

(Collected and Communicated.)

HOSPITAL MEETINGS.

Italian Hospital.—The annual general meeting will be held at the Italian Embassy on January 29th, at 4.30.

The Duke of Cambridge has promised to preside at a festival dinner in aid of the North London Hospital for Consumption, to be held at the Hotel Cecil on May 19th.

H.R.H. the Duke of York has promised to preside at the 83rd anniversary festival of the Royal Caledonian Asylum, which will be held on Monday, May 9th, at the Hotel Metropole.

Mr. Chisholm Williams will lecture at the City Orthopædic Hospital on Tuesday, February 1st, at half-past five p.m., on Rachitic Deformities.

In a private letter received from Bombay, one of the doctors engaged in plague duty says, "Often when we pay our visits of inspection to native homes we find the head of the household sharpening a huge knife wherewith he has pledged his word with neighbours to stab the first doctor sahib who enters his household. In one village we visited, over 3,000 of the inhabitants had disappeared and gone to various hiding-places, whence they emerged so soon as we had finished our tour. Of course we were helpless in the matter. We knew the population was missing, but could not scour the country and conduct our physical examinations by force. It would have been like an Afridi expedition. So we returned with our work only half done."

The Federation of Working Men's Social Clubs, of which the Duke of Westminster is president, has arranged to hold a series of discussions on the subject of the London hospitals. The first meeting will be held on Wednesday, February 16th, at a quarter-past eight p.m. at the London Chamber of Commerce, St. Botolph House, Eastcheap, when the subject under discussion will be, "The better organisation of the sources of support to hospitals on the part of the working classes, and in particular on the part of working men's clubs, and similar bodies. Express reference to be made under this head to the Hospital Saturday Fund." The subjects to be discussed at the following meetings embrace reform in the out-patient department, the position of provident dispensaries in connection with hospitals, and the central board scheme.

Eastbourne enjoys the distinction of the lowest death-rate in England. The stringent measures enforced against pig-styes, backyard poultry, and other rear premise nuisances are worthy of imitation.

The Council of the Sanitary Institute have accepted an invitation from the Lord Mayor and City Council of Birmingham to hold its seventeenth congress and exhibition in that city in September next.

A festival dinner in aid of the East London Hospital for children is to be held on Tuesday, March 22nd, at the Whitehall Rooms, Mr. B. L. Cohen, M.P., L.C.C., in the chair.

A festival dinner in aid of the North London or University College Hospital will be held at the Hotel Metropole on Wednesday, February 2nd, the Duke of Bedford presiding.

It is to be hoped the Maidstone inquiry will include a house-to-house searching sanitary inspection, for in the poorer districts in many of the houses there is absolutely no water supply to the w.c.'s, and rear premises need an Augean cleansing.

Following a recent example, Mrs. Laurie has forwarded to the secretary of the British Home for Incurables, Streatham, a cheque for £1,000, to establish the "Menella, In Memorium" bed in the home, and in the hope that others may be induced to help the charity in a similar manner.

At the Gwelo (Matabeleland) Hospital the fever tents are now pitched, and form a picturesque addition to the landscape. During January, February, and March fever is most prevalent, and the tents, capable of holding between 20 to 30 patients, are kept usually very full. The rainy season and the fever season go together. Miners and mounted police during the rains get their clothes and blankets drenched, and they cannot light a fire to dry them, and frequently are wet through for a week at a time, with the natural result that they are "down with fever."

At the Mansion House, on Friday, the 14th inst., a meeting of the Council of the Metropolitan Hospital Sunday Fund was held for the purpose of electing committees and officers for the present year and transacting general business. At the opening of the proceedings the Lord Mayor had not arrived, and the chair was taken by Lord Stamford. The General Purposes Committee was first elected, to consist of the Lord Mayor (President *ex-officio*), Sir S. H. Waterlow (Vice-President), the Chief Rabbi, Archdeacon Sinclair, and 18 other gentlemen. The Committee of Distribution of the past year was re-elected. The Special Committee for surgical appliances, orders, and hospital letters was ordered to consist of any two members of the Council acting with the secretary. Sir E. H. Currie and Mr. R. B. Martin, M.P., were re-elected honorary secretaries, Mr. H. N. Custance reappointed secretary, and Messrs. W. H. Pannell and Co. were once more reappointed honorary auditors. This concluded the business. Those present were Sir A. T. Watson, Sir Savile Crossley, Sir E. H. Currie, Lord F. G. Fitzroy, Mr. S. Holland, M.P., Captain Cundy, Messrs. A. Thorne, R. Grey, H. Hoskier, J. Mackrell, Macnamara, A. L. Cohen, and G. Drummond, Canons Graham and Ingram, the Revs. J. B. Burnaby, W. H. Barlow, A. Harland, and C. H. Grundy, and Sir H. Burdett.

